# Transforming Growth Factor Beta and Epithelial to Mesenchymal Transition Alter Homologous Recombination Repair Gene Expression and Sensitize BRCA Wild-Type Ovarian Cancer Cells to Olaparib

**DOI:** 10.3390/cancers15153919

**Published:** 2023-08-01

**Authors:** Cai M. Roberts, Mehida Rojas-Alexandre, Ruth E. Hanna, Z. Ping Lin, Elena S. Ratner

**Affiliations:** 1 Department of Pharmacology, Midwestern University, 555 31st St., Downers Grove, IL 60515, USA; 2 Department of Obstetrics, Gynecology, and Reproductive Sciences, Yale University School of Medicine, 15 York St., New Haven, CT 06510, USA

**Keywords:** ovarian cancer, PARP inhibitors, epithelial to mesenchymal transition, drug response

## Abstract

**Simple Summary:**

Olaparib is a PARP inhibitor that is currently the standard treatment for ovarian cancer. However, its use is largely confined to tumors carrying a mutation in the BRCA1 or BRCA2 genes. Our study sought to identify additional ovarian cancer cell populations sensitive to olaparib. TGFβ has been well characterized as a driver of epithelial to mesenchymal transition (EMT), a process whereby epithelial cancer cells alter their adhesion molecules and gain the ability to migrate and invade. We hypothesized that the cytokine TGFβ would alter DNA repair mechanisms that render wild-type ovarian cancer cells sensitive to olaparib. We used two pairs of epithelial and mesenchymal ovarian cancer cell lines to probe DNA repair and olaparib response. Our findings suggest that some populations of metastatic cancer cells may be vulnerable to olaparib or other therapies targeting DNA repair.

**Abstract:**

Epithelial ovarian cancer (EOC) remains the most lethal gynecologic malignancy, largely due to metastasis and drug resistant recurrences. Fifteen percent of ovarian tumors carry mutations in BRCA1 or BRCA2, rendering them vulnerable to treatment with PARP inhibitors such as olaparib. Recent studies have shown that TGFβ can induce “BRCAness” in BRCA wild-type cancer cells. Given that TGFβ is a known driver of epithelial to mesenchymal transition (EMT), and the connection between EMT and metastatic spread in EOC and other cancers, we asked if TGFβ and EMT alter the susceptibility of EOC to PARP inhibition. Epithelial EOC cells were transiently treated with soluble TGFβ, and their clonogenic potential, expression, and function of EMT and DNA repair genes, and response to PARP inhibitors compared with untreated controls. A second epithelial cell line was compared to its mesenchymal derivative for EMT and DNA repair gene expression and drug responses. We found that TGFβ and EMT resulted in the downregulation of genes responsible for homologous recombination (HR) and sensitized cells to olaparib. HR efficiency was reduced in a dose-dependent manner. Furthermore, mesenchymal cells displayed sensitivity to olaparib, cisplatin, and the DNA-PK inhibitor Nu-7441. Therefore, the treatment of disseminated, mesenchymal tumors may represent an opportunity to expand the clinical utility of PARP inhibitors and similar agents.

## 1. Introduction

Epithelial ovarian cancer (EOC) remains the leading cause of death among gynecologic malignancies, with approximately 20,000 new cases and over 13,000 deaths anticipated in 2023 [1]. Most EOC cases are diagnosed late, typically at stage III or IV, and despite generally favorable responses to chemotherapy, the disease often recurs following treatment. Such recurrences are often widely metastatic and resistant to first line therapeutic agents. Five-year survival for stage III/IV disease hovers around 30% [2]. Therefore, new treatment paradigms are urgently needed.

Fifteen percent of EOC tumors carry mutations or deletions of BRCA1 or BRCA2 [3], a defect in homologous recombination (HR) that renders these tumor cells vulnerable to synthetic lethality induced by inhibitors of poly (ADP-ribose) polymerase (PARP). PARP is required for efficient repair of single strand breaks (SSBs) via base excision repair (BER). SSBs left unchecked will become double strand breaks (DSBs) upon replication of DNA. Defective HR due to BRCA1/2 loss results in cells depending heavily on PARP-mediated repair such as BER, thus a blockade of both HR and BER leads to synthetic lethality [4,5]. The PARP inhibitor (PARPi) olaparib has become the standard therapy for ovarian tumors with BRCA mutations, in the settings of initial or recurrent maintenance therapy [6]. However, resistance can develop due to reversion of BRCA to wild-type (WT) [7,8]. Thus, whether reverted or simply never mutated, most EOC tumors are not currently considered candidates for olaparib therapy.

Recent work from our group has investigated the use of olaparib in combination with additional agents for the treatment of BRCA-WT cancer. In particular, we have found that triapine, an inhibitor of ribonuclease reductase, can sensitize EOC cells to olaparib, topoisomerase inhibitors, or platinum chemotherapy to kill cancer cells in the presence of WT BRCA1/2 [9,10]. We also characterized an additional ribonucleotide reductase inhibitor, DB4, which showed a similar effect [11]. Further work found that combining olaparib with triapine and the vascular endothelial growth factor receptor (VEGFR) inhibitor cediranib was effective against BRCA-WT and PARPi-resistant EOC xenografts [12].

These studies demonstrate the potential of PARP inhibition in BRCA WT tumors, but in the present study we sought to identify intrinsic conditions that would sensitize BRCA WT cells to PARPi without the need for combination therapy. We therefore turned our attention to transforming growth factor beta (TGFβ), a well characterized cytokine and known oncogene that has been associated with tumor progression and epithelial to mesenchymal transition (EMT) [13]. TGFβ signals via serine/threonine kinase type receptors to activate a number of downstream signals, including SMAD-dependent and -independent pathways [14,15,16]. Recent work has linked TGFβ to DNA damage response. TGFβ-deficient murine mammary cells display an impaired response to DNA damage from ionizing radiation [17]. In a mouse model of prostate cancer, radiation increased TGFβ expression [18,19]. TGFβ also contributes to bone marrow failure in Fanconi anemia, while its inhibition promoted HR over the alternative pathway non-homologous end joining (NHEJ) [20]. In 2014, Liu et al. found that TGFβ could induce a state resembling BRCA1/2 loss, often termed “BRCAness”, by downregulating DNA repair factors in breast cancer, thus sensitizing breast cancer cells to PARPi [21]. However, whether TGFβ plays a similar role in EOC is unknown.

In addition to TGFβ signaling, we investigated the impact of EMT as a whole on DNA repair and responses to PARPi. EMT involves the loss of cell adhesion molecules binding epithelial cells in a monolayer and the upregulation of new cell surface proteins that favor motility and survival without attachment [22,23,24,25]. As such, EMT and its drivers such as TWIST1 have long been associated with the metastatic spread of solid tumors, including ovarian [22,26,27]. The relationship of EMT to drug response, however, is complicated. In EOC, CD44+ cancer stem cells are inherently chemoresistant and are epithelial in character, while their CD44- mesenchymal progeny are fast-dividing and therefore chemosensitive [28]. However, across many other tumor types, EMT is correlated with stemness [29,30]. Among mesenchymal ovarian cancer cells, EMT correlates with drug resistance, and the reduction of EMT factors such as TWIST1 has been shown to sensitize such cells to chemotherapy [31,32]. Here, we investigate the effect of EMT on HR and olaparib response.

We demonstrate that either transient TGFβ signaling or the acquisition of the mesenchymal phenotype via EMT can reduce HR factor expression in EOC cells, leading to sensitivity to olaparib. Given the prevalence of metastatic disease amongst recurrent EOC, the ability to efficiently eliminate mesenchymal cells shows great promise in combatting advanced disease. When combined with our prior studies on combination therapies, our findings support the continued investigation and clinical use of PARPi in BRCA WT EOC tumors.

## 2. Materials and Methods

### 2.1. Chemicals and Reagents

Cisplatin was obtained from Calbiochem/EMD Millipore (Billerica, MA, USA). Olaparib and cediranib were obtained from Selleck Chemicals (Houston, TX, USA). TGFβ and LY2109761 were obtained from Cayman Chemical (Ann Arbor, MI, USA). Triapine was synthesized in the Ratner laboratory at Yale University School of Medicine.

### 2.2. Cell Lines

PEO1 and PEO4 cells were a gift from Dr. Peter Glazer at Yale University. These lines were derived from a single patient at different points in the course of treatment for ovarian carcinoma [33]. PEO1 is a BRCA2-mutant, chemosensitive line with mesenchymal morphology. PEO4 was derived from recurrence following platinum-based chemotherapy. It carries a BRCA2 reversion to WT, is chemoresistant, and displays epithelial morphology. Both lines were maintained in DMEM with 10% FBS and 1% penicillin/streptomycin. R182 and TARA-R182 cells (also referred to as CD44+ OCSC-1 and CD44− OCC-1, respectively) were a gift from Dr. Gil Mor at Wayne State University and have been described previously [34,35]. Both lines were maintained in RPMI-1640 with 10% FBS, and 1% each of penicillin/streptomycin, non-essential amino acids, sodium pyruvate, and HEPES. SKOV-3 cells (HTB-77) were purchased from the American Type Culture Collection (ATCC, Manassas, VA, USA) and maintained in McCoy’s 5A or RMPI-1640 with 10% FBS and 1% penicillin/streptomycin. SKOV-3-DR-GFP cells expressing a reporter cassette were derived from WT SKOV-3 and have been described previously [9,10,11]. All cells were grown in a humidified 37 °C incubator at 5–6% CO_2_.

### 2.3. Clonogenic Assays

PEO1 and PEO4 cells were seeded in triplicate wells of six-well plates and allowed to adhere for 24 h, at densities of 250, 500, 1000, 2500, and 5000 cells per well. R182 and TARA-R182 (hereafter, TARA) cells were plated in single wells at 500 cells per well. Cells were then treated with olaparib (0.31–5 µM), TGFβ (5 µM), and/or LY2109761 (0.5 µM) and incubated undisturbed in standard cell culture conditions for 10–21 days as indicated. At the conclusion of this window, the medium was removed, and cells were fixed and stained in 0.5% crystal violet in 50% methanol. Excess dye was washed out with water and plates were air dried. Images were obtained of PEO1 and PEO4 cells using a GelDoc imager, and colonies were counted using QuantityOne analysis software (both from Bio-Rad, Hercules, CA, USA). For R182 and TARA cells, where colony counting was impossible due to the growth pattern of cells, a standard digital camera image was obtained of each plate.

### 2.4. Western Blotting

Cell samples were lysed in RIPA buffer or SDS Lysis Buffer (1% SDS, 10 mM Tris HCl, pH7.4) and protein concentrations determined via DC Protein Assay (Bio-Rad) or BCA Assay (Thermo Fisher Scientific, Waltham, MA, USA). Equal masses of protein were run on Mini-PREOTEAN TGX gels (Bio-Rad) and transferred to nitrocellulose or PVDF membranes. Membranes were blocked in 5% milk in phosphate- or Tris-buffered saline with 0.5% Tween-20 (PBST or TBST) for >40 min at room temperature. Membranes were incubated with primary antibodies in 5% BSA in PBST/TBST overnight at 4 °C. Membranes were then rinsed in PBST/TBST, incubated with HRP-conjugated secondary antibodies for 1–2 h at room temperature, and rinsed again with PBST/TBST. Blot images were obtained using enhanced chemiluminescent substrate (Bio-Rad or Denville Scientific, South Plainfield, NJ, USA) and a ChemDoc imager from Bio-Rad or G:BOX imager from Syngene (Frederick, MD, USA). Beta actin and HSC-70 were used as loading controls. Anti-BRCA1 (D-9), anti-Rad51 (H-92), anti-β-Actin (C4), and anti-HSC70 (K-19) antibodies were purchased from Santa Cruz Biotechnology (Dallas, TX, USA). Anti-BRCA2 (A303-434A) was from Bethyl Laboratories (Fortis, Waltham, MA, USA). Anti-fibronectin (MS-1351), anti-snail (3879S), and anti-N-Cadherin (MA1-2002) were from ThermoFisher (Waltham, MA, USA). Anti-vimentin (3390S), anti-E-Cadherin (5296S), anti-AKT (4691S), and anti-pAKT (4058S) were from Cell Signaling Technology (Danvers, MA, USA).

### 2.5. MTS Assay

A total of 1000–3000 cells per well were plated in 96-well plates and allowed to adhere for 24 h. The following day, cells were treated with cisplatin, olaparib, cediranib, or Nu-7441 and incubated in the presence of the drug and 100 μL medium per well for 72 h. Wells containing media only and no cells were used to establish background. After 72 h, 20 μL MTS (CellTiter 96 AQueous One Solution, Promega, Madison, WI, USA) was added to each well and cells were incubated for a further 2 h. Absorbance at 490 nm was then read using a plate reader. Background was subtracted and absorbance was converted to percent viability compared with controls.

### 2.6. HRR Assay

SKOV-3-DR-GFP cells were pretreated with 5 ng/mL or 20 ng/mL TGFβ for 24 h before being transiently transfected with the empty vector pcDNA/Neo (Thermo Fisher) or the I-SceI endonuclease expression vector pCBASceI using the TransFast reagent (Promega, Madison, WI, USA) according to the manufacturer’s protocol. Five hours after transfection, cells were retreated with 5 ng/mL TGFβ, 20 ng/mL TGFβ, or 0.75μM triapine for 48 h. Cells were trypsinized and analyzed for the percentage of GFP-positive cells by flow cytometry using a LSR II flow cytometer and FlowJo software (Version 10.4; BD Biosciences, East Rutherford, NJ, USA).

### 2.7. Quantitative Reverse Transcription PCR

Total RNA was obtained from R182 and TARA cell pellets using the Total RNA Kit from IBI Scientific (Dubuque, IA, USA). RNA yield and quality was assayed using absorbance spectra on a NanoDrop 2000 (Thermo Fisher). cDNA was reverse transcribed using the qScript cDNA synthesis kit from Quantabio (Beverly, MA, USA). Quantitative PCR was run on an Applied Biosystems Quantstudio 5 qPCR machine (Thermo Fisher), using PerfeCTa SYBR Green FastMix and Low ROX (Quantabio) in 10 μL reactions. PCR was run for 40 cycles, followed by melt curve analysis. Beta actin was used as a housekeeping gene. Relative gene expression levels were calculated using the 2^−ΔΔCt^ method. Primers were obtained from Integrated DNA Technologies (Coralville, IA, USA), and sequences were as follows: BRCA2-Fd, 5′-AAAACGTTGAGCTGTTGCCA-3′; BRCA2-Rv, 5′-TGTGTTTTGGTTGAATTGTACCTT-3′; RAD51 Fd, 5′-CCAGACCCAGCTCCTTTACC-3′; RAD51 Rv, 5′-CACTGCGACACCAAACTCATC-3′; BRCA1 Fd, 5′-GGCTATCCTCTCAGAGTGACATTT-3′; BRCA1 Rv, 5′-GCTTTATCAGGTTATGTTGCATGGT-3′; Actin Fd, 5′-TTCCTGGGCATGGAGTCC-3′; and Actin Rv, 5′-CAGGTCTTTGCGGATGTCC-3′.

### 2.8. Wound Healing Assay

Cells were plated in 6-well plates and incubated 24 h to allow for the formation of a complete monolayer. Where indicated, cells were treated with 5 ng/mL TGFβ for one hour prior to scratching. Scratches were made using a sterile P200 pipet tip. Images of the wound were obtained immediately and after 24 h.

### 2.9. Statistics

MTS data are shown as mean ± S.D. qPCR data incorporate standard deviation in the calculation of fold change ranges. Clonogenic data are shown as mean ± S.E. for each condition. Comparisons of two samples (i.e., R182 vs. TARA) were performed using Student’s *t* test. HRR assay was analyzed using one-way ANOVA with Dunnett’s test for multiple comparisons. All analyses were completed in GraphPad Prism 9. A *p* value < 0.05 was considered statistically significant. All experiments were repeated at least twice.

## 3. Results

### 3.1. TGFβ Induces Mesenchymal Morphology in Ovarian Cancer Cells

**Figure 1:**
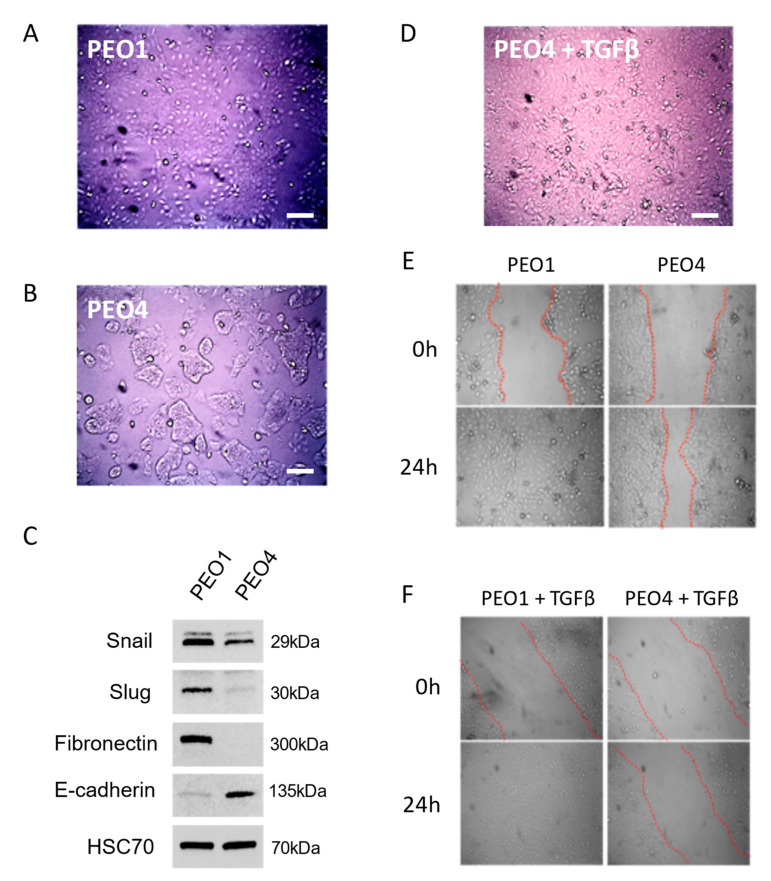
Differences in mesenchymal marker expression and morphology in PEO1, PEO4, and TGFβ-treated cells. (**A**). Micrograph of PEO1 cells, showing monolayer growth. (**B**). Micrograph of PEO4 cells, showing growth in islands. (**C**). Representative Western blot of epithelial and mesenchymal markers in PEO1 and PEO4. HSC-70: loading control. (**D**). Following 5 ng/mL TGFβ treatment, PEO4 cells adopt PEO1-like morphology. (**E**). Wound healing assays show that PEO1 rapidly migrates to close a wound, while PEO4 has a slower rate of healing. (**F**). Treatment with 5 ng/mL TGFβ does not appreciably change migratory potential in either line. Scale bars in (**A**,**B**,**D**), 100µm.

We first chose to examine the effects of TGFβ on a pair of ovarian cancer cell lines derived from a single patient. PEO1 has mesenchymal-like morphology when grown as a monolayer (Figure 1A) and is a BRCA2 mutant and therefore sensitive to olaparib. PEO4 was derived from a recurrence and has a reversion of BRCA2 to WT, rendering it resistant to olaparib. Interestingly, its morphology is more epithelial (Figure 1B) and its growth rate slower than PEO1, suggesting an epithelial-like subpopulation acquired a novel BRCA2 mutation that allowed it to outcompete a faster-growing but drug-responsive majority of tumor cells. The epithelial versus mesenchymal status of the two lines was verified by Western blot, which showed elevated expression of snail, slug, and fibronectin in PEO1 compared to PEO4. Conversely, E-Cadherin was elevated in PEO4 compared to PEO1 (Figure 1C and Supplementary Figure S1). When we treated PEO4 cells with the EMT inducing cytokine TGFβ, we observed that they adopt a more fibroblast-like morphology and ultimately resemble PEO1 (Figure 1D). To further examine mesenchymal character, we next assayed migratory potential of the two lines with and without TGFβ treatment. PEO1 exhibited higher migratory ability than PEO4, as shown by wound healing assays (Figure 1E). However, treatment of PEO4 with TGFβ was not sufficient to increase wound healing (Figure 1F).

### 3.2. TGFβ Alters Expression of EMT Markers in PEO1 and PEO4

**Figure 2:**
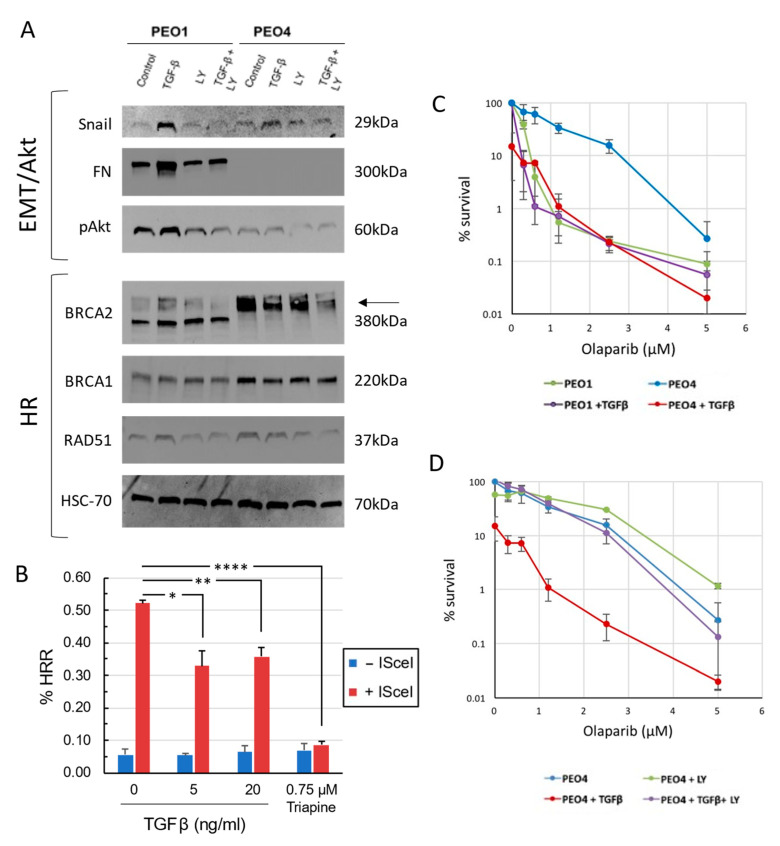
TGFβ reduces HR and sensitizes PEO4 cells to olaparib. (**A**) Top: TGFβ upregulates snail in both PEO1 and PEO4 cells, and fibronectin (FN) and phospho-Akt in PEO1. TGFβ receptor inhibitor LY2109761 (LY) abrogates these changes and lowers pAkt in both lines. Bottom: TGFβ decreases BRCA2, BRCA1, and RAD51 expression in PEO4. HSC-70: loading control. Representative blot image shown. (**B**) Percent GFP positive SKOV-3 cells following the treatments shown in HR reporter assay. ISceI-expressing cells treated with 5 or 20 ng/mL TGFβ or 0.75 µM triapine were compared to untreated ISceI-expressing controls. *, *p* < 0.05; **, *p* < 0.01; ****, *p* < 0.0001 by one-way ANOVA with Dunnett’s multiple comparisons test. (**C**) Clonogenic assay demonstrates that PEO4 is olaparib-resistant, while PEO4 + TGFβ is sensitive and shows a similar response to PEO1 with or without TGFβ. (**D**) Clonogenic assay shows LY2109761 reverses the effect of TGFβ on olaparib response in PEO4. Cells were incubated undisturbed for 14–21 days prior to quantitation.

We next examined the expression of epithelial and mesenchymal markers in PEO1 and PEO4 cells, with and without TGFβ treatment. Treatment with 5 ng/mL TGFβ resulted in the increased expression of snail in both lines, and upregulated fibronectin (FN) in PEO1. Co-treatment with the TGFβ receptor inhibitor LY2109761 (0.5 µM) abrogated these changes, showing that TGFβ signaling was responsible for the observed increase in marker expression (Figure 2A and Supplementary Figure S2). TGFβ also upregulated phosphorylated Akt in PEO1, while LY2109761 reduced phospho-Akt in both lines. PEO4 did not express fibronectin, and TGFβ was not sufficient to induce fibronectin expression in this line (Figure 2A and Supplementary Figure S2). Together, these data indicate that TGFβ induces a partial EMT in PEO4 while further upregulating mesenchymal genes and promoting growth and proliferation in the already mesenchymal PEO1.

### 3.3. TGFβ Downregulates HR Proteins in BRCA2 WT Cells and Sensitizes Them to Olaparib

Based on prior reports of TGFβ influencing the expression of DNA repair factors [19,20], we also asked whether HR pathway factors would be differentially expressed in our lines and following TGFβ treatment. BRCA1 and BRCA2 were downregulated in PEO1 compared to PEO4, while expression of RAD51 was similar between the two lines (Figure 2A and Supplementary Figure S2). The treatment of PEO4 with TGFβ decreased the expression of BRCA1, BRCA2, and to a lesser degree RAD51, but this effect was not seen in PEO1 (Figure 2A and Supplementary Figure S2). Treatment with LY2109761 rescues this effect on BRCA1, although BRCA2 expression following combination treatment remains low (Figure 2A and Supplementary Figure S2). To determine if loss of HR protein expression translates to loss of HR efficiency, we utilized an HR reporter system built in the BRCA WT cell line SKOV-3 [9,10,11]. Briefly, SKOV-3-DR-GFP cells were treated with TGFβ or the ribonuclease reductase inhibitor triapine as a positive control. Cells were then transfected with I-SceI nuclease, which cleaves the reporter cassette. Successful repair of the break via HR leads to the expression of GFP, which we measured using flow cytometry. Pretreatment with 5 or 20 ng/mL TGFβ significantly reduced the number of GFP+ cells following I-SceI expression, indicating that TGFβ pretreatment impaired HR, though not to the extent seen with triapine (Figure 2B and Supplementary Figure S3). Given the induction of “BRCAness” in PEO4 cells following TGFβ treatment, we next asked whether the response to olaparib would be impacted. Clonogenic assays showed that, as expected, PEO4 cells were substantially more resistant to olaparib compared to PEO1. Furthermore, while TGFβ treatment had no effect on the response of PEO1, TGFβ-treated PEO4 cells were sensitized to olaparib, and showed a dose response similar to that of BRCA2 mutant PEO1 (Figure 2C). LY2109761 completely blocked this effect, indicating that the change in response was due to TGFβ signaling (Figure 2D).

### 3.4. EMT Also Sensitizes Ovarian Cancer Cells to PARP Inhibition

**Figure 3:**
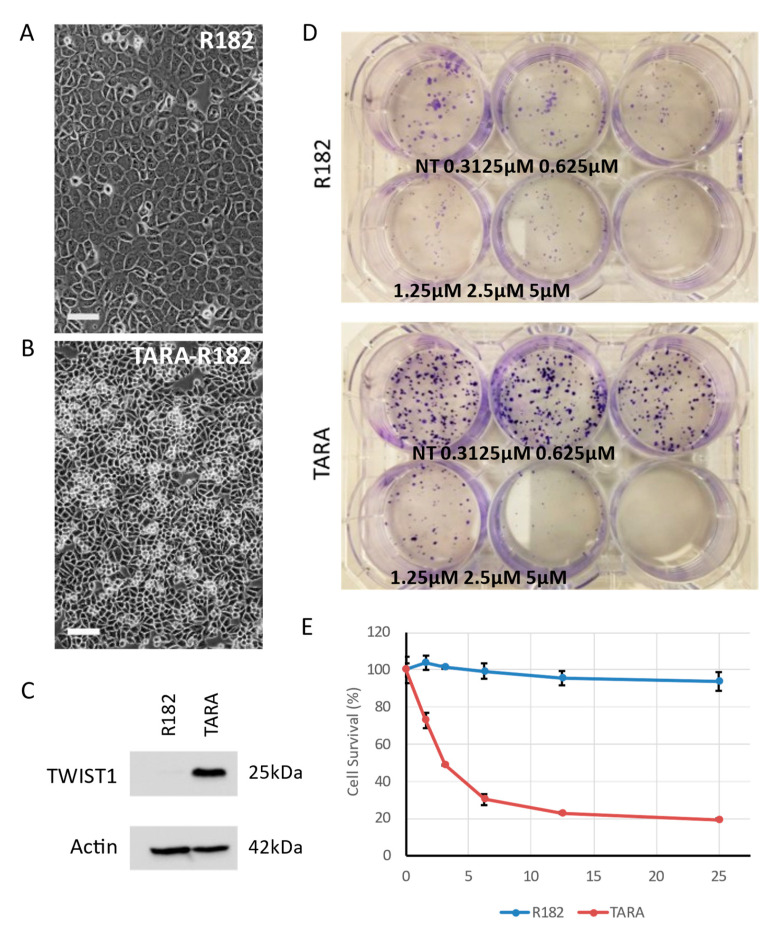
TARA cells are more sensitive to olaparib than R182. (**A**) R182 cells show cobblestone-like epithelial morphology. (**B**) TARA-R182 cells show slightly angular morphology reminiscent of PEO1, as well as the ability to grow on top of the monolayer (round cells). Scale bars, 100 µm. (**C**) TARA-R182 cells are mesenchymal as shown by the expression of the EMT marker TWIST1, as compared to the epithelial, TWIST1 negative R182. Representative blot shown. (**D**) Top: Clonogenic assay of R182 cells treated with the indicated doses of olaparib. Increasing dose yields smaller colonies, but little change in colony number. Bottom: The same assay in TARA cells shows TARA cells are more sensitive to doses ≥1.25 μM. Cells were incubated undisturbed for ten days prior to staining. (**E**) MTS assay measuring cell proliferation shows robust dose response in TARA cells treated with olaparib, while R182 is unaffected. Error bars, S.D. of technical triplicates; representative graph of three independent experiments shown.

As TGFβ treatment of PEO4 produced only a partial and transient EMT phenotype, we next sought to confirm our findings in EOC cells that had undergone EMT and retained their mesenchymal character indefinitely. To do this, we utilized the R182 and TARA cell line models first developed and described by Mor and colleagues [28,34,35,36]. R182 cells exhibit classic epithelial “cobblestone” morphology (Figure 3A). TARA cells, which were derived from in vitro EMT of R182 cells, are smaller (Figure 3B), faster growing, and express mesenchymal markers, including the EMT driver TWIST1 (Figure 3C and Supplementary Figure S4). We first performed clonogenic assays using R182 and TARA cells treated with varying doses of olaparib. The growth pattern of R182 was not conducive to accurate colony counts; however, images of stained cells clearly show that, for doses up to 5 μM, olaparib reduced the growth rate of R182 but had li”tle ’ffect on the number of colonies formed (Figure 3D). In contrast, the treatment of TARA cells with as little as 0.625 μM olaparib produced a notable reduction in colony number, while a 5 μM dose completely abrogated TARA growth (Figure 3D). Cell proliferation assays comparing R182 and TARA cells also showed minimal inhibitory effects of olaparib in R182 cells but a clear dose response in TARA cells (Figure 3E). Thus, the pattern of olaparib sensitivity in mesenchymal cells holds for this model.

### 3.5. EMT Alters HR Gene Expression

**Figure 4:**
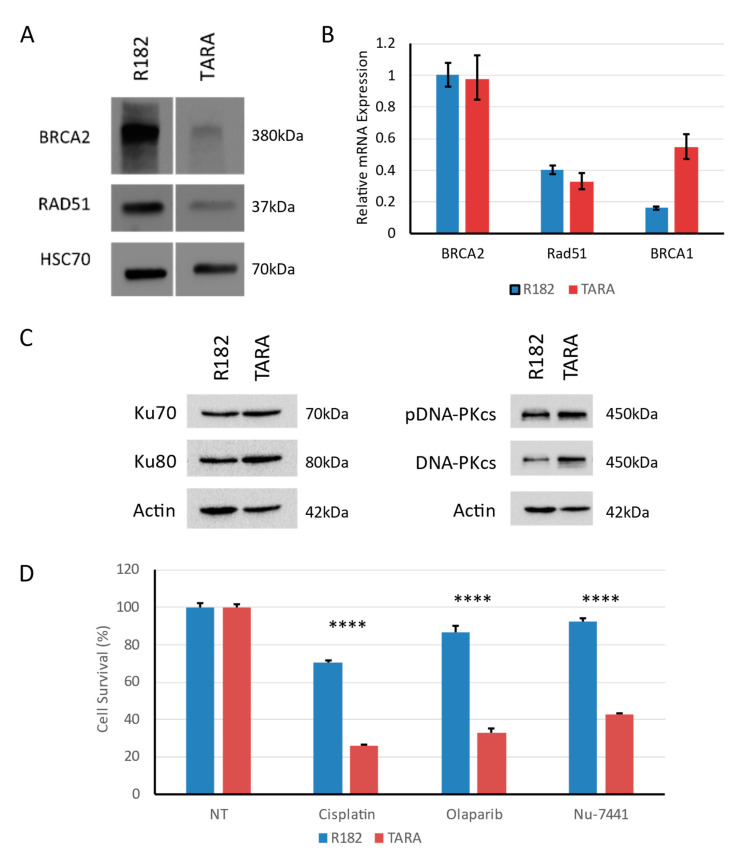
Changes in DNA repair and drug susceptibility in TARA cells. (**A**) Western blot shows that expression of BRCA2 and RAD51 are reduced in TARA vs. R182. HSC-70, loading control. Representative blot shown. (**B**) qPCR shows that BRCA2 and RAD51 mRNA levels are not substantially changed between R182 and TARA cells, while BRCA1 mRNA expression trends higher in TARA. Error bars, range of fold changes; representative graph of three independent experiments shown. (**C**) Western blot shows levels of Ku70, Ku80, and DNA-PKcs proteins are elevated in TARA vs. R182. Actin, loading control. Representative blots shown. (**D**) MTS assay of R182 and TARA cells treated with indicated drugs. Cisplatin, 10 μM; olaparib, 25 μM; Nu-7441, 10 μM. Error bars: S.D. of technical triplicates. Representative graph of three independent experiments shown. **** *p* < 0.0001.

We next sought to determine whether changes in olaparib response in TARA versus R182 cells were the result of changes in HR gene expression. Western blot showed, that compared to R182, TARA cells expressed lower levels of BRCA2 and RAD51 protein (Figure 4A and Supplementary Figure S4). However, qPCR demonstrated no significant change in mRNA expression between R182 and TARA for either of these genes, suggesting a post-transcriptional mechanism of BRCA2 and RAD51 loss (Figure 4B). Interestingly, BRCA1 mRNA was upregulated in TARA cells compared to R182, but this was not sufficient to give rise to olaparib resistance (Figure 3).

### 3.6. EMT Induces an Alternate DNA Repair Pathway and Opens an Additional Therapeutic Window

Given the fast growth rate of TARA cells despite the lower expression of HR proteins, we hypothesized that alternate means of DNA repair may be upregulated. The most common alternate pathway for the repair of DSBs is canonical non-homologous end joining (c-NHEJ). In this process, Ku70 and Ku80 form a complex with the catalytic subunit of DNA-dependent protein kinase (DNA-PKcs) at DNA breaks, leading to the recruitment of DNA ligase IV and XRCC4 and the sealing of the break [37]. We found that, in TARA cells, the expression of Ku70 and Ku80 were upregulated compared to R182, as were the levels of total and phosphorylated DNA-PKcs (Figure 4C and Supplementary Figure S5). Since TARA cells are increasingly reliant on c-NHEJ for DSB repair, we next asked whether the targeting of c-NHEJ could represent an additional therapeutic strategy against mesenchymal cells. We treated R182 and TARA cells with 10 μM Nu-7441, an inhibitor of DNA-PK. As expected, approximately 60% of TARA cells were killed by this dose, while less than 10% of R182 cells were (Figure 4D). However, at this dose, NU-7441 can also inhibit PI3K and mTOR, so we cannot be sure that the inhibition of c-NHEJ alone is responsible for TARA cell growth inhibition, especially in the absence of exogenous DNA damage [38].

### 3.7. TGFB1 and BRCA2 Are Inversely Correlated in Ovarian Carcinoma Patient Samples

**Figure 5:**
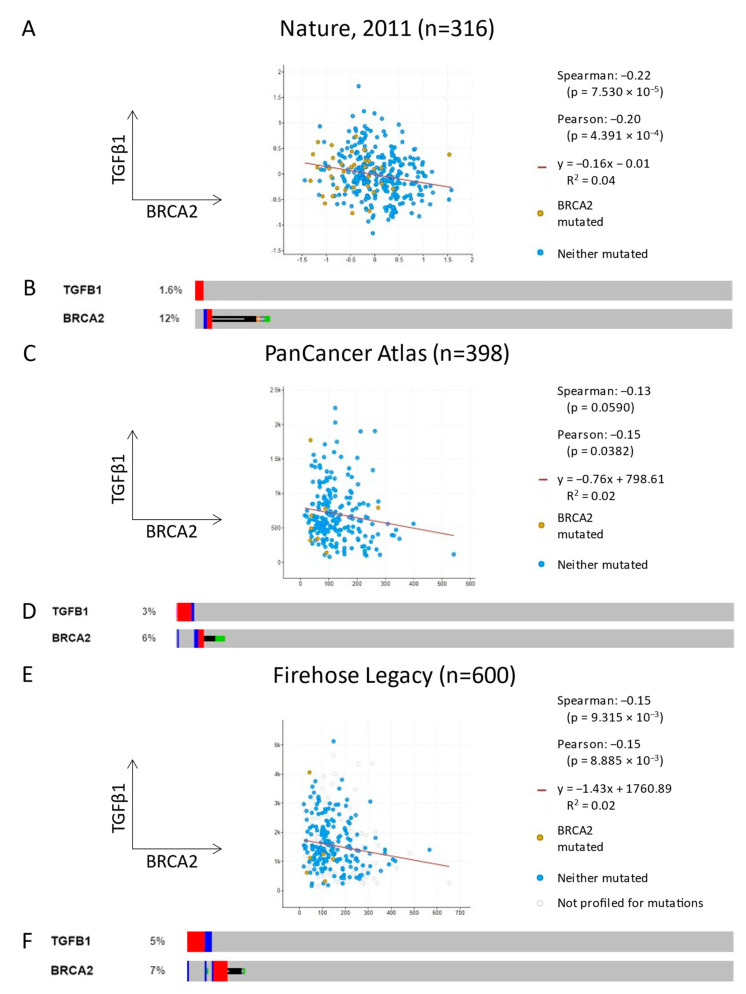
cBioPortal analysis of TCGA ovarian cancer datasets supports inverse relationship between TGFβ and BRCA2. (**A**,**C**,**E**) mRNA expression data of *TGFB1* vs. *BRCA2* for the indicated datasets. In each case, expression of the two genes is negatively correlated. (**B**,**D**,**E**) Gene amplification (red), deletion (blue), or mutation (black/orange/green) of the indicated genes. Grey represents samples with no change. *TGFB1* and *BRCA2* are never co-amplified, and in only one case are both deleted (**F**).

In order to determine whether our findings translated to patient tumors, we explored three TCGA ovarian cancer datasets using cBioPortal [39,40]. The expression of *TGFB1* and *BRCA2* were negatively correlated amongst samples evaluated for the expression and alteration of both genes across the Nature 2011, PanCancer Atlas, and Firehose Legacy datasets. Pearson correlation scores were −0.20, −0.15, and −0.15, respectively, and the relationship achieved statistical significance for all datasets. Spearman correlation scores were similar and achieved significance for all but the PanCancer Atlas dataset (*p* = 0.059) (Figure 5A,C,E). In addition, *TGFB1* and *BRCA2* were never amplified together in any sample in any dataset (Figure 5B,D,F, red sections). These data suggest that the relationship between TGFβ and BRCA2 that we see in our cell lines is reflected in patient tumors.

## 4. Discussion

Despite much research and the advent of PARPi-based therapy, ovarian cancer remains a highly deadly disease, with more than 13,000 deaths anticipated in the United States in 2023 [1]. Chief among the clinical challenges of EOC are recurrence and therapy resistance, which often coincide with widely metastatic disease. Only a minority of EOC tumors carry a BRCA mutation or other HR defect, meaning most patients are not normally candidates for PARPi-based therapy [3]. However, we have recently made the case for opening a therapeutic window for the use of olaparib in BRCA WT tumors by inducing “BRCAness”, for example, by using olaparib in combination with triapine [10,12]. In the present study, we sought additional cancer cell populations that would be vulnerable to PARPi. Recently, it was demonstrated that TGFβ sensitized breast cancer cells to PARPi by inducing “BRCAness” [21]. TGFβ has been well characterized as a driver of EMT, a developmental program that is reactivated in cancers in response to a stressful environment and gives rise to metastatic spread [22,41,42,43]. Given the association between metastatic progression and mortality in EOC, we asked whether TGFβ and EMT would impact DNA repair and PARPi response in EOC.

We first established the EMT status, DNA repair state, and drug response profile of PEO1 and PEO4 cells. PEO1 cells display mesenchymal-like character and sensitivity to olaparib on the basis of a BRCA2 mutation. Interestingly, PEO4 shows altered morphology with growth in islands, increased E-Cadherin expression, and loss of fibronectin expression, suggesting a more epithelial phenotype (Figure 1C). By restoring BRCA2 expression and function, PEO4 cells developed resistance to olaparib. However, the treatment of PEO4 cells with the pro-EMT cytokine TGFβ was sufficient to reduce levels of BRCA2, RAD51, and BRCA1 protein and sensitize PEO4 cells to olaparib (Figure 2). Notably, the dose response for these cells post-TGFβ was comparable to that of PEO1, a line that lacks functional BRCA2. Moreover, an inhibitor of TGFβ signaling was sufficient to abrogate changes in both gene expression and drug response, demonstrating that the effect is TGFβ-dependent. Furthermore, TGFβ upregulated phospho-Akt in PEO1 cells, while inhibition of TGFβ signaling reduced Akt activation. This is in alignment with prior work showing that EMT can impact the expression and activation of Akt, and that Akt promotes survival and drug resistance [32,44].

As the PEO4 line was established at a later time than their PEO1 counterparts, it is possible that in addition to a reversion of BRCA2 to WT, PEO4 cells have undergone a mesenchymal to epithelial transition (MET) in vivo. EMT is often thought of as an irreversible process; it is more likely that tumor cells undergo a transient or incomplete EMT that allows them to retain some epithelial characteristics and seed secondary sites [45,46,47]. In the case of this model, PEO4 may have just shifted further back toward an epithelial state. Alternatively, the features of PEO4 may suggest that a subpopulation of drug-resistant epithelial cells were selected for by initial rounds of platinum-based chemotherapy. This interpretation is consistent with work that identified epithelial cancer stem cells that were resistant to therapy and could repopulate a tumor [28]. It is also consistent with the concept of tumor heterogeneity, which recognizes that tumors are mosaics of several lineages of cancer cells within a single patient [48]. Tumor heterogeneity represents a barrier to therapy due to the challenge of killing all lineages. The need to address multiple populations of cells may mean that combinations of therapeutics with different mechanisms of action (including targeting DNA repair) will be more successful than monotherapy or multiple lines of monotherapy.

Following our studies of PEO1 and PEO4, we went on to ask whether an additional model, representing EMT as opposed to transient TGFβ exposure, would show a similar trend. We therefore made use of the cell lines R182 and TARA-R182, also referred to as OCSC-1 and OCC-1, respectively [34,35]. These syngeneic cell lines have previously been shown to be epithelial-like and mesenchymal-like, respectively, and we reiterate this via the analysis of morphology and expression of the EMT marker TWIST1 (Figure 3). As hypothesized, mesenchymal TARA cells were more sensitive to olaparib compared to their R182 forebears, as shown by both clonogenic and MTS cell quantitation assays (Figure 3). Moreover, TARA cells express lower levels of BRCA2 and RAD51 protein, despite little to no change in mRNA levels (Figure 4). Interestingly, BRCA1 mRNA is upregulated in TARA versus R182, though this is not sufficient to cause olaparib resistance. These findings are not consistent with the results of two studies in breast cancer. In the first, TGFβ treatment of breast cancer cells led to “BRCAness” through the downregulation of a different set of HR genes, namely ATM, MSH2, and BRCA1 [21]. Wu et al. also showed that, in triple negative breast cancer, EMT factors slug and snail led to repression of BRCA1 expression via recruitment of the demethylase LSD1 and direct binding of the *BRCA1* promoter [49].

In order to place our results within the context of clinical ovarian cancer, we analyzed existing TCGA datasets and asked whether expression of *TGFB1* and *BRCA2* were related. As shown in Figure 5, we found an inverse relationship in each of three datasets, supporting our findings here. Previous work has also shown that the mesenchymal marker TWIST1 correlates with poor survival in an additional patient cohort [50]. Loss of HR capacity in mesenchymal cells may therefore represent an opportunity to improve outcomes for patients with mesenchymal tumors, although much work remains to be completed.

Finally, we hypothesized that due to loss of HR throughput, TARA cells would upregulate the c-NHEJ pathway to compensate. Indeed, we showed that TARA cells have an increased expression level of factors required for the c-NHEJ pathway, including Ku70, Ku80, and DNA-PKcs (Figure 4). Based on this observation, we assayed the response of TARA versus R182 cells to Nu-7441, an inhibitor of DNA-PK. As seen for olaparib, TARA cells were more sensitive to Nu-7441 than R182. As noted earlier, the dose of Nu-7441 used is sufficient to impact additional targets, including PI3K and mTOR, and inhibition of these pathways may well contribute to TARA cell death. Furthermore, the development of NU-7441 itself as a putative drug was halted due to concerns with solubility and thus bioavailability, meaning an alternative such as the DNA-PK and PI3K dual inhibitor KU-0060648 may be a better candidate for further development [51,52]. However, these results represent early data in support of broadening DNA-repair-targeted therapy in ovarian cancer, and in cancers at large. Walker et al. recently showed that the DNA repair landscape of ovarian tumors can predict survival, further supporting this assertion [53]. The same study found that HR and c-NHEJ defects were mutually exclusive, in agreement with our findings in TARA cells, and suggested that DNA repair status could be an important therapeutic consideration in non-ovarian tumors. Agents targeting DNA repair are a newer addition to our anti-cancer arsenal, and while they have faced challenges during development, they hold great promise for new treatment avenues [51,54].

**Figure 6:**
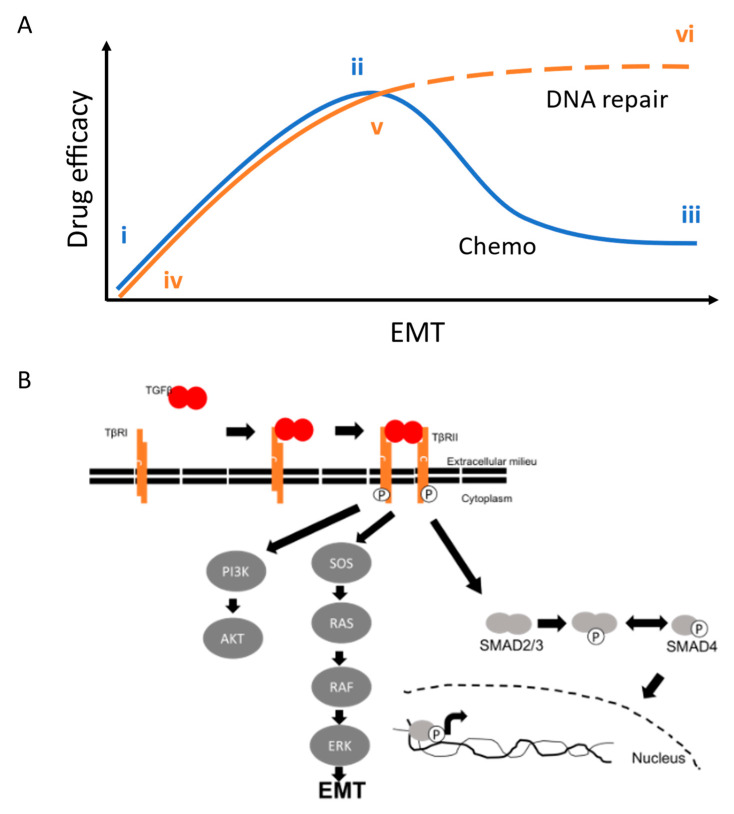
Models of EMT and TGFβ effects on tumor signals and drug responses. (**A**) Relationship between EMT status and therapy response. (**i**–**iii**). As shown here and in other studies, EMT gives rise to fast-dividing, chemosensitive cells that go on to develop secondary resistance to cytotoxic chemotherapy (blue curve). (**iv**,**v**). We show here that EMT also leads to the increased efficacy of DNA-repair-targeted drugs, such as olaparib and Nu-7441 (orange curve). (**vi**) Further work is needed to determine whether chemoresistant cells retain sensitivity to DNA-repair-targeted agents, either alone or in combination (orange dashes). (**B**) TGFβ signaling pathways. Canonical TGFβ binds to its receptor, leading to the activation of Akt, SMAD proteins, and EMT. Additional signals connecting EMT and HR remain to be elucidated.

The role of EMT in determining drug responses is complicated, perhaps more so in EOC than in other tumor types. Nevertheless, we have developed a schematic to explain the relationship between EMT and drug responses in EOC over the course of the disease (Figure 6). Unlike many cancers, in which mesenchymal character correlates with stem-like phenotype [29,30], we and others have observed that CD44+ epithelial cells serve as cancer stem cells in EOC [27,28]. These stem cells, represented by the line R182, are inherently resistant to chemotherapy (Figure 6A, i), but give rise to fast-dividing mesenchymal-like cells that are chemosensitive (i.e., TARA; Figure 6A, ii). Amongst mesenchymal cells, acquired resistance can manifest as EMT and tumor evolution progress (Figure 6A, iii). Mesenchymal-like OVCAR8 cells displaying resistance to cisplatin were resensitized via the inhibition of TWIST1, leading to reduced Akt activation under cell culture conditions and reduced tumor growth in vivo [31,32]. Similarly, Craveiro et al. showed that OCSC1-F2 cells, derived from R182 via in vivo rather than in vitro EMT, are initially sensitive to paclitaxel. However, despite mice carrying F2 tumors going into remission following paclitaxel treatment, the tumors recur and are paclitaxel-resistant [34]. In the current study, we sought to determine whether the same pattern of drug responses as cells undergo EMT extends to PARP inhibitors, and more broadly, DNA-repair-targeting therapies. In epithelial-like R182 and PEO4 cells, the response to olaparib was low (Figure 6A, iv) while in mesenchymal TARA and PEO1 cells, the response was high (Figure 6A, v). PEO1 is expected to be sensitive to olaparib on the basis of its BRCA2 mutation, and TARA cells exhibit a similar trend due to their downregulated HR proteins. However, it remains unknown whether sensitivity to DNA-repair-directed therapies, including olaparib or Nu-7441, either alone or in combination, will remain effective in advanced disease that has become resistant to platinum or taxane therapy (Figure 6A, vi).

It is also unknown which signals downstream from TGFβ mediate its effect on DNA repair. A promising candidate is the complex of SMAD2/3, and preliminary data (not shown) suggest that a knockdown of SMAD2 can oppose the effects of TGFβ on HR. However, our data suggest that EMT, not just TGFβ signals, can impact HR, and the EMT program may be activated via non-SMAD factors (Figure 6B). Therefore, our future work will include transcriptomic analyses of our cell line models to determine the mechanisms underlying the phenotypes we report here. Effective leveraging of synthetic lethality in BRCA WT cells will depend on identifying novel targets within these pathways and designing drugs to inhibit them, and our future work will take the first steps toward these translational goals.

## 5. Conclusions

**Figure 7:**
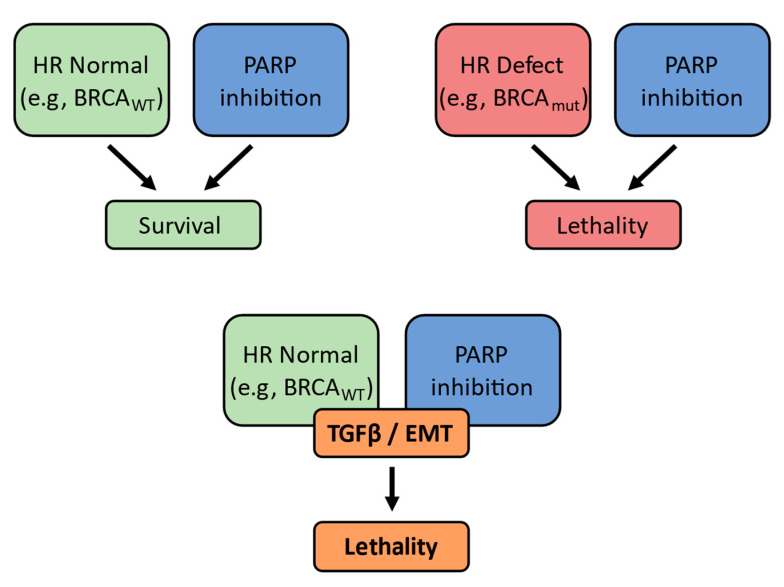
Schematic of overall findings. TGFβ signaling of EMT can give rise to olaparib lethality even in the presence of WT HR proteins.

Our data clearly demonstrate that TGFβ treatment and the acquisition of a mesenchymal phenotype in EOC cells results in the downregulation of factors necessary for the HR pathway of DNA repair, and thus the sensitization of these cells to PARP inhibition (Figure 7). Because mesenchymal, metastatic cells common to EOC recurrence can manifest resistance to first-line chemotherapy, the addition of PARPi and other DNA-repair-targeting drugs to our arsenal, even for BRCA WT tumors, may represent a step forward in combating recurrent EOC and ultimately improving patient survival.

## Data Availability

All data generated or analyzed during this study are included in this published article and its Supplementary Materials.

## Supplementary Materials

The following supporting information can be downloaded at: https://www.mdpi.com/article/10.3390/cancers15153919/s1, Figure S1: Full western images for Figure 1C. Figure S2: Full western images for Figure 2A. Figure S3: Flow cytometry data for Figure 2B. Figure S4: Full western images for Figure 3C and Figure 4A. Figure S5: Full western images for Figure 4C.
