# Supplementary material for: Transforming Growth Factor Beta and Epithelial to Mesenchymal Transition Alter Homologous Recombination Repair Gene Expression and Sensitize BRCA Wild-Type Ovarian Cancer Cells to Olaparib
Source: Cancers (Basel). 2023 Aug 1;15(15):3919. doi: 10.3390/cancers15153919 (PMC10417836; doi:10.3390/cancers15153919)
Supplement: Supplementary file 1 [file cancers-15-03919-s001.zip › cancers-2525749-supplementary (1).pdf]

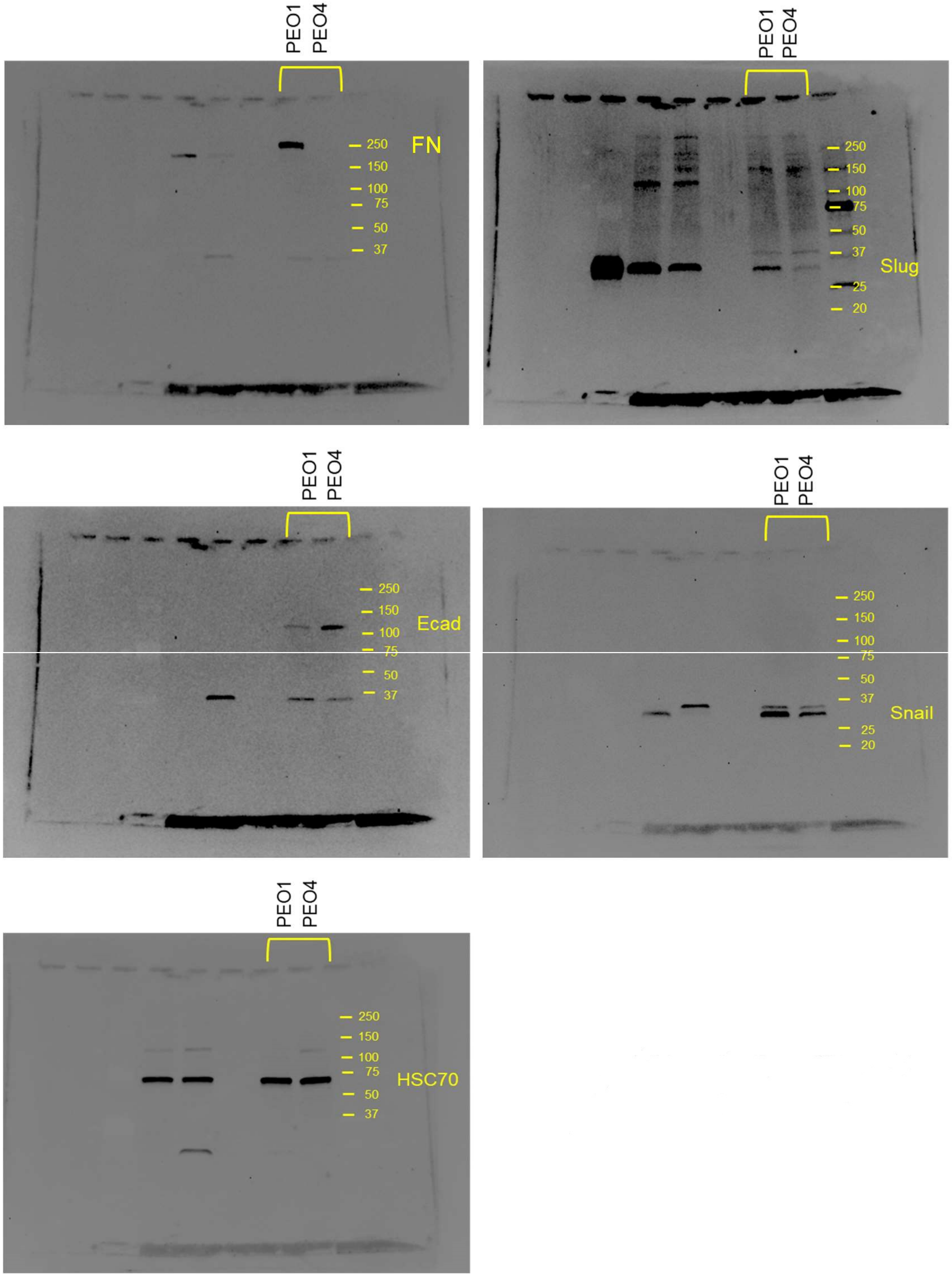

Figure S1. Full western blot images for Figure 1C

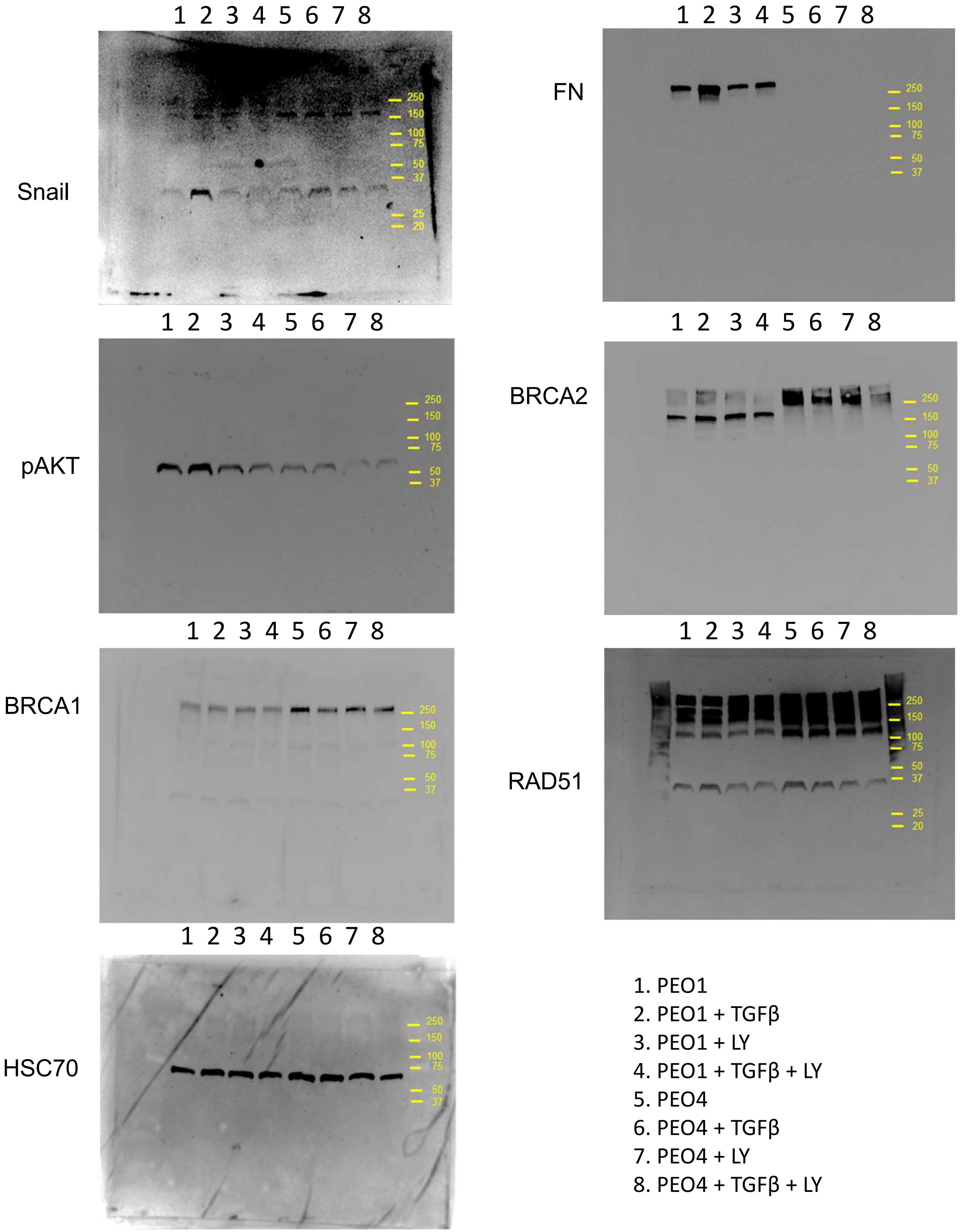

1. PEO1
2. PEO1 + TGFβ
3. PEO1 + LY
4. PEO1 + TGFβ + LY
5. PEO4
6. PEO4 + TGFβ
7. PEO4 + LY
8. PEO4 + TGFβ + LY

Figure S2. Full western blot images for Figure 2A

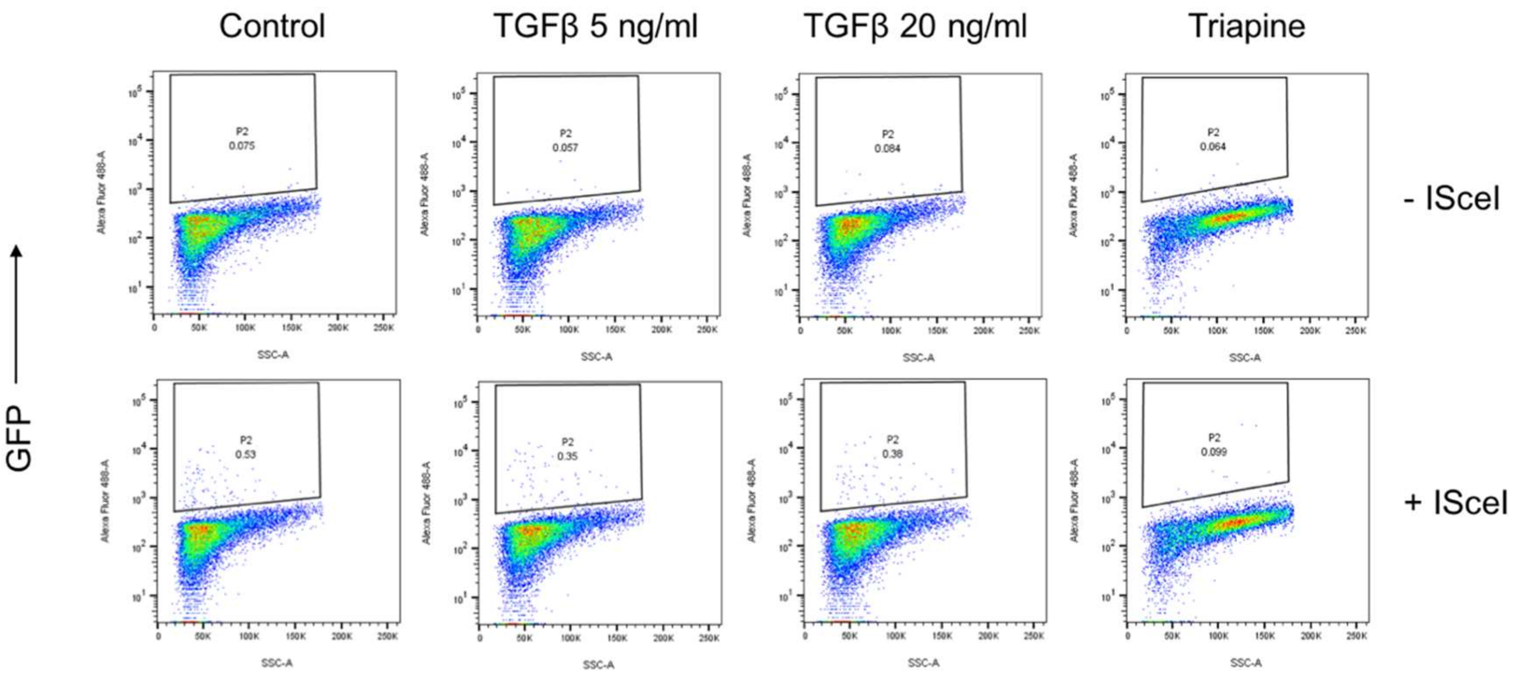

Figure S3. Representative flow cytometry data for experiment shown in Figure 2B.

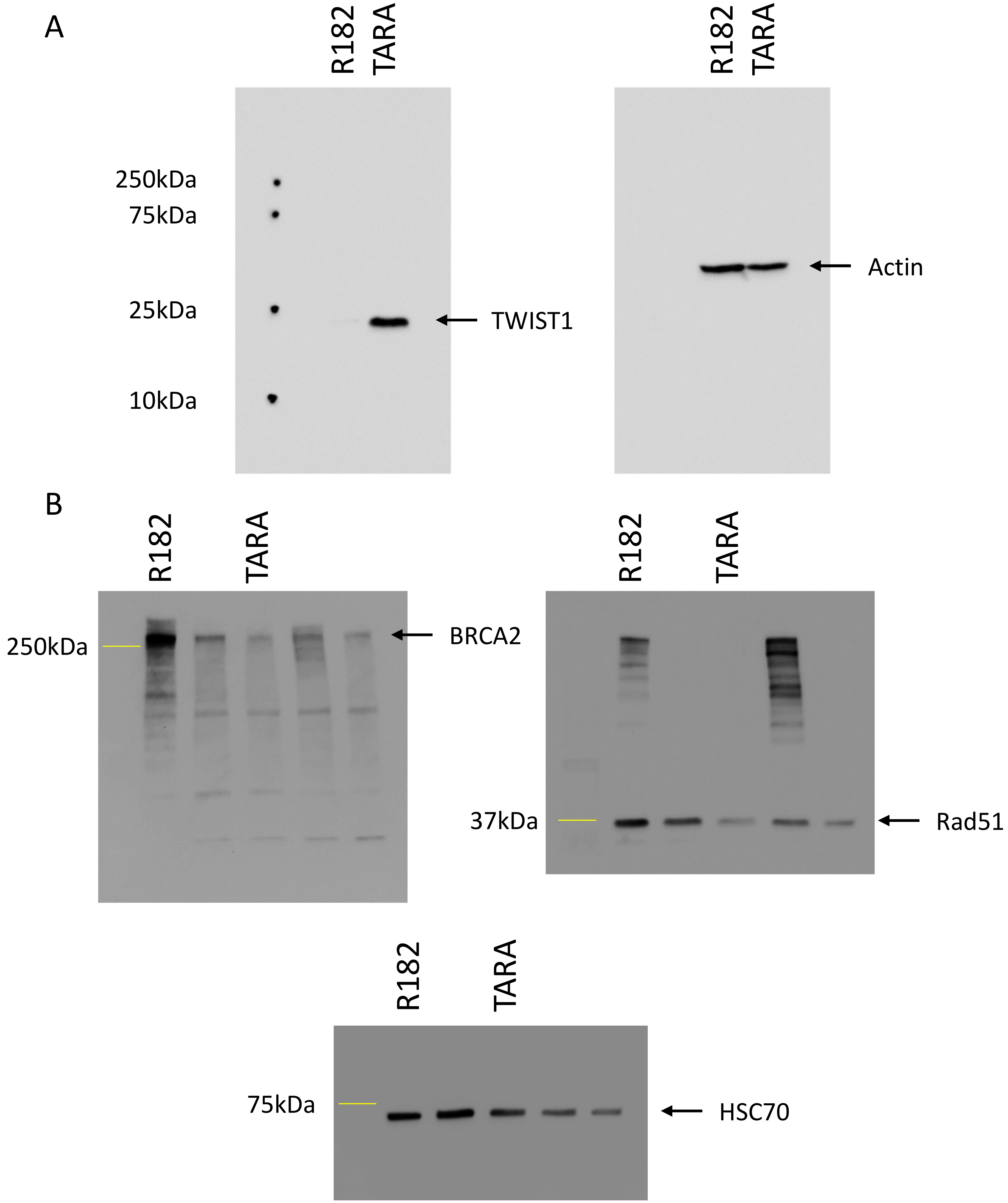

Figure S4. Full western blot images for data shown in **A.** Figure 3C and **B.** Figure 4A.

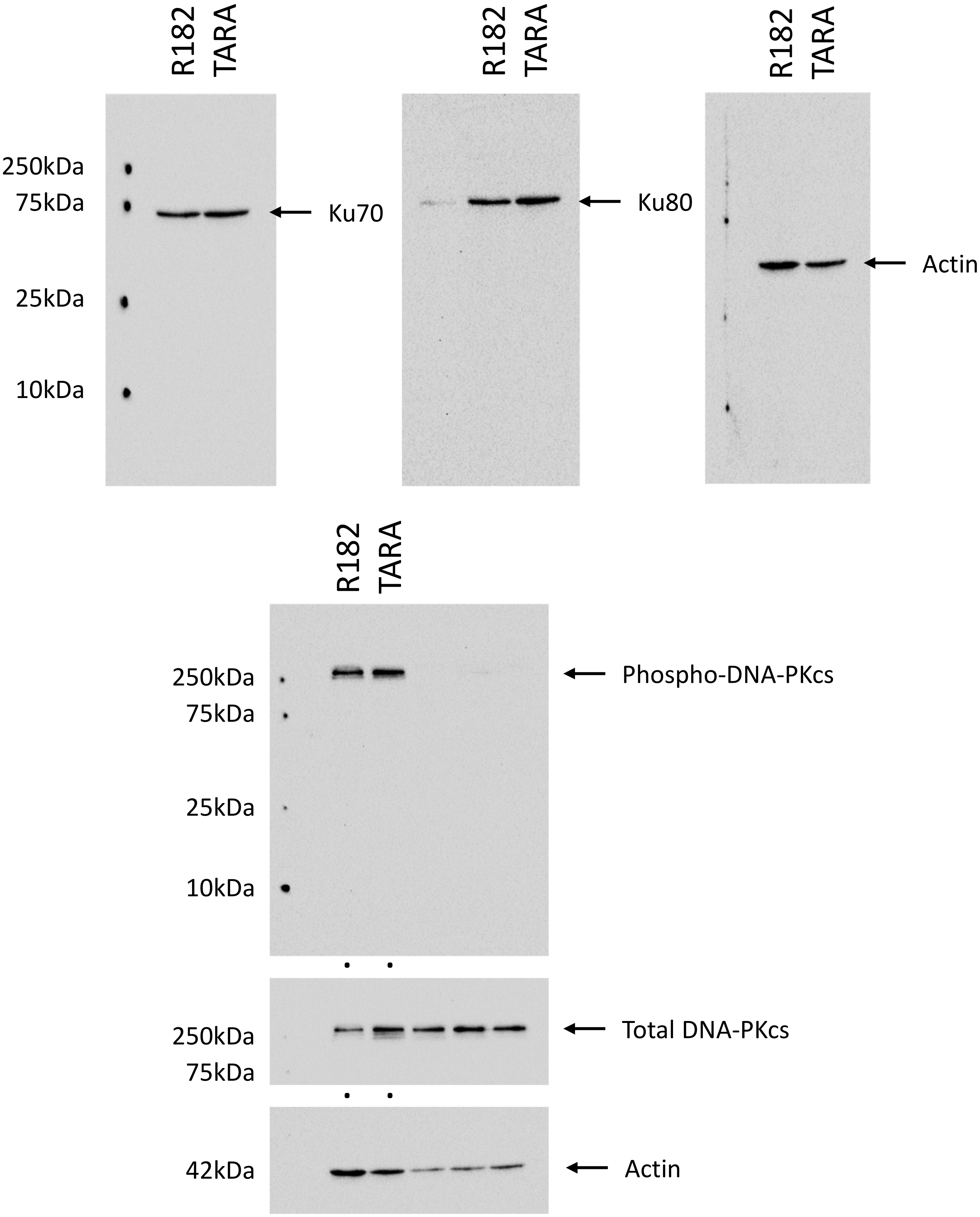

Figure S5. Full western blot images for data shown in Figure 4C.
